# Bladder-Exstrophy-Epispadias-Complex risk and C677T polymorphism in MTHFR gene: case-control study among Indian children

**DOI:** 10.1186/1755-8166-7-S1-P22

**Published:** 2014-01-21

**Authors:** Abid Ali, Rajeev Kumar Pandey, Sukanya Gayan, Minu Bajpai

**Affiliations:** 1 Department of Pediatric Surgery, All India Institute of Medical Sciences, New Delhi-110029, India

## Background

Bladder exstrophy is a congenital anomaly in which part of the urinary bladder is present outside the body. It is rare in occurrence but the frequency is increasing very rapidly. 5, 10-methyltetrahydrofolate reductase (MTHFR) enzyme, which catalyzes the synthesis of 5-methylenetetrahydrofolate and C677T polymorphism in MTHFR, shows a significant role with a thermolabile enzyme and decreased specific MTHFR activity. The objective of the present investigation was to study the case-control association between C677T polymorphism in relation to Bladder-Exstrophy-Epispadias-Complex.

## Materials and methods

For the present study, a total of 50 patients classified as Bladder-Exstrophy-Epispadias-Complex & cloacal exstrophy patient and 50 healthy school going children (as control) were recruited to participate in this study. Genomic DNA was extracted from peripheral blood lymphocytes by using commercially available kits. Genotypes of the MTHFR C677T polymorphisms were detected by polymerase chain reaction–restriction fragment length polymorphism (PCR-RFLP).

## Results

Frequency distributions of genotypes and combined genotypes were obtained. The overall distribution of the C677T genotype was found to be significantly associated with cloacal exstrophy but not with epispadias as compared to the controls.

## Conclusions

Genotyping of 50 patient cases and 50 controls revealed a significant association between C677T polymorphism and Bladder-Exstrophy.

